# Atoll-scale patterns in coral reef community structure: Human signatures on Ulithi Atoll, Micronesia

**DOI:** 10.1371/journal.pone.0177083

**Published:** 2017-05-10

**Authors:** Nicole L. Crane, Peter Nelson, Avigdor Abelson, Kristin Precoda, John Rulmal, Giacomo Bernardi, Michelle Paddack

**Affiliations:** 1 Department of Biology, Cabrillo College, Aptos, California, United States of America; 2 Oceanic Society, Ross, California, United States of America; 3 One People One Reef, Santa Cruz, California, United States of America; 4 H. T. Harvey & Associates, Bldg D, Los Gatos, California, United States of America; 5 Department of Zoology, Tel Aviv University, Ramat Aviv, Israel; 6 Marine Studies Institute, University of Sydney, Sydney, New South Wales, Australia; 7 Ulithi Falalop Community Action Program, Yap, Federated States of Micronesia; 8 Department of Ecology and Evolutionary Biology, University of California, Santa Cruz, California, United States of America; 9 Santa Barbara City College, Santa Barbara, California, United States of America; Department of Agriculture and Water Resources, AUSTRALIA

## Abstract

The dynamic relationship between reefs and the people who utilize them at a subsistence level is poorly understood. This paper characterizes atoll-scale patterns in shallow coral reef habitat and fish community structure, and correlates these with environmental characteristics and anthropogenic factors, critical to conservation efforts for the reefs and the people who depend on them. Hierarchical clustering analyses by site for benthic composition and fish community resulted in the same 3 major clusters: cluster 1–oceanic (close proximity to deep water) and uninhabited (low human impact); cluster 2–oceanic and inhabited (high human impact); and cluster 3–lagoonal (facing the inside of the lagoon) and inhabited (highest human impact). Distance from village, reef exposure to deep water and human population size had the greatest effect in predicting the fish and benthic community structure. Our study demonstrates a strong association between benthic and fish community structure and human use across the Ulithi Atoll (Yap State, Federated States of Micronesia) and confirms a pattern observed by local people that an ‘opportunistic’ scleractinian coral (*Montipora* sp.) is associated with more highly impacted reefs. Our findings suggest that small human populations (subsistence fishing) can nevertheless have considerable ecological impacts on reefs due, in part, to changes in fishing practices rather than overfishing per se, as well as larger global trends. Findings from this work can assist in building local capacity to manage reef resources across an atoll-wide scale, and illustrates the importance of anthropogenic impact even in small communities.

## Introduction

It has been more than a decade since large-scale and long-term studies began to empirically document the global deterioration of coral reef communities from processes such as large-scale bleaching, overfishing, high nutrient loads, crown-of-thorns starfish (COTs) invasions, and other perturbations [1]. Given the dependence of many coastal human communities on reefs for critical resources such as food and coastal protection, and the high variation in reef structure across both small and large spatial scales [2–4], there is an increasing need for data on reefs across multiple spatial and temporal scales in order to address pressing issues such as food security and reef resiliency [5–9]. It is increasingly clear that much of the deterioration, including eutrophication, physical damage, and bleaching, is due to human impacts, either directly or indirectly, and often in combination with ecological factors [10–13]. However, the dynamic relationship between reefs and the people who utilize them is poorly understood [14].

Coral reef assemblages vary across large geographic scales, as well as in response to more localized oceanographic factors including currents, salinity, temperature, exposure, and access to biotic recruitment pools [2,15]. Human use of coral reefs also has profound effects on reef community structure, and, in combination, these factors can drive observable patterns in coral reef assemblages over a variety of spatial scales [7,9]. Few studies have characterized coral reefs across atoll-wide scales in regions where human populations are small but extractive activities have been prevalent for thousands of years (but see [4,16,17]). Ulithi Atoll, Federated States of Micronesia, is a system that includes spatial variation in reef type due to both abiotic and biotic factors, including human influence.

Coral reef declines are evident in systems close to large human communities and those affected by commercial fishing operations, but also on seemingly ‘pristine’ reefs utilized by relatively small numbers of people for subsistence purposes only [9,14,18,19]. Quantifying human activities as drivers of coral reef patterns can be challenging, but identifying human signatures in those patterns over multiple scales is crucial to understanding and mitigating ecological impacts, even in remote locations where population density is low and there is limited or no industrialization. In these systems, subtle changes in fishing techniques and traditional practices, rather than burgeoning populations and industrial-scale fishing, may be partially driving change [20,21].

Large tracts of reef exist in remote locations, and data from these systems are often limited or lacking entirely [22]. The policies and practices of local communities in many of these regions determine how reefs and resources are managed, and local people need information to build local capacity for successful long-term planning [23]. This is the case for Ulithi Atoll, where humans have been utilizing reef resources for millennia [24]. The historically stable human populations on these isolated islands suggest that reef resources were sufficiently abundant in the past.

Despite almost continuous resource extraction from the reefs of Ulithi for over 2000 years [24,25], contemporary surveys of the area indicate that the Atoll reefs support a diverse and comparatively healthy reef community [26,27]. More recently, however, people of Ulithi have been expressing concern regarding the state of their reefs, dwindling fish populations, a relatively “new” (within the past ca. 50 years) species of *Montipora* coral, which is reportedly spreading over the reef at several locations, and a “poisonous” *Rhodactis* corallimorph reef (John Rulmal Jr, personal observation) [28,29].

Reef changes raise food security, as well as management and conservation, concerns for these communities [30]. Large-scale impacts such as climate change and ocean acidification, and more local anthropogenic impacts over the past 50–70 years such as military activities during World War II, the introduction of ‘new’ fishing methods (e.g. spear guns and cast nets), the advent of small motorboats, and the loss of traditional management frameworks, have likely all contributed to changes in reef health and fish abundance on Ulithi. These patterns need to be better understood in order to document climate change impacts and the effects of endemic human drivers (including resource extraction and management), as well as to facilitate mitigation planning, adaptation, and local capacity building.

Ulithi Atoll therefore presents a unique opportunity to study an isolated social-ecological system whose ecosystem services have been compromised, at least in part due to subsistence level extraction activities in the relatively recent past despite continuous use for over 2000 years [25]. This study characterizes atoll-scale patterns in shallow coral reef habitat and fish community structure by identifying patterns and correlations between environmental characteristics, human communities, and biological systems on Ulithi Atoll. Specifically, we addressed: 1) reef fish and benthic community structure variability across the Atoll, 2) characteristics that define reef types, and 3) factors influencing this variation.

## Materials and methods

### Ethics statement

We received specific permission to sample reefs from Philip Paiy, Chief of Falalop, Lipipi Clan, representing Council of Ten (CO-X), Ulithi Atoll. We also received a formal invitation to conduct the research from the Council of Tamol (Branch of Yap legislature representing the outer islands), and received stated support from Yap Marine Resources. We had formal meetings with the Governor’s office (Yap) and the Legislature to brief them on the work. Finally, we received on-site permission from village Chiefs (or designee) prior to each sampling visit before any in-water work was conducted. All data and findings were shared with each village on every island.

No institutional Animal Care and Use committee approval was needed; no animals were disturbed, collected or sacrificed. However, all sampling procedures were reviewed and approved by the local communities as part of obtaining permission to complete this research.

### Study location

**Fig 1:**
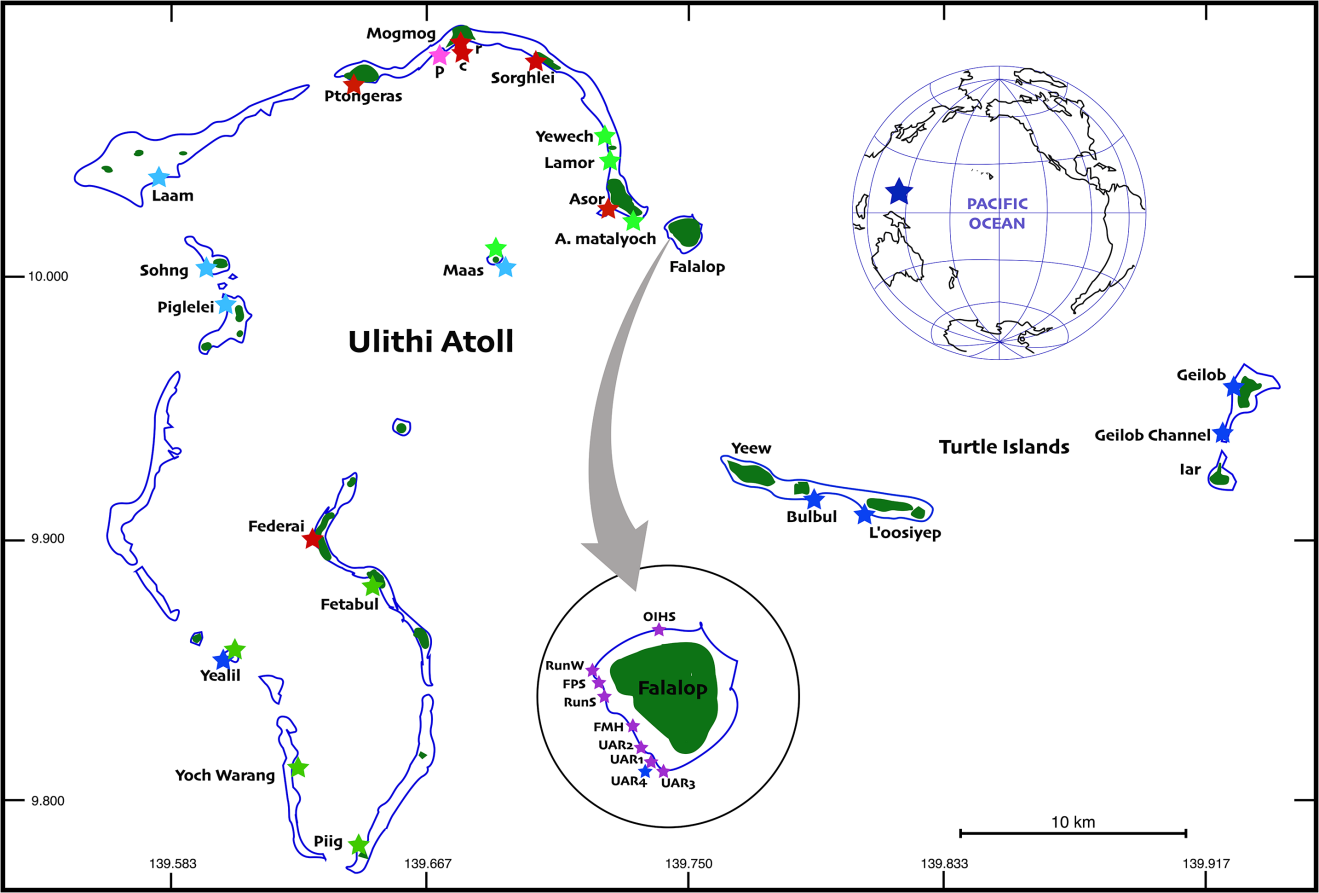
Study site locations (colored stars) at Ulithi Atoll and at neighboring Turtle Islands. Sites were classified according to relative levels of impact from human activities and exposure to NE trade winds and the prevailing swell direction: Red—Inhabited, Lagoonal, Green—Uninhabited, Lagoonal, Blue—Uninhabited, Oceanic, Purple—Inhabited, Oceanic. The location of Ulithi Atoll in the western Pacific Ocean is shown in the inset (blue star, upper right).

The Ulithi lagoon encompasses approximately 500 km^2^ making it one of the largest atolls on Earth (Fig 1). The Atoll consists of ca. 44 low-lying (max. elevation ca. 10 m) islets and islands (total land area ca. 4.3 km^2^). The diversity of reef types (and associated biodiversity) is high due to both physical factors and human settlements [27].

Permanent residents of Ulithi number about 900, with approximately another 350 people from other neighboring islands living on the Atoll during the school year (August-May). The 4 inhabited islands are Falalop (which lies just outside the main Atoll) with a population of between 500–700 people, MogMog (Northern part of the Atoll), with a population of approximately 150, Asor (North-East part of the Atoll), with a population of approximately 70, and Federai to the southeast, with a population of approximately 150 (Fig 1). The populations of these islands have remained relatively stable, with the exception of Falalop, which has increased by about 450 people since 1949 (this is largely due to the building of an airstrip during World War II, and the construction of the Outer Islands High School in 1961) [24].

### Site selection

During three consecutive years (2012–2014), we sampled reef fishes, benthic composition, coral morphology, and coral distribution at 32 sites throughout the Atoll and associated nearby islands (Fig 1, Table 1). We selected sites across a broad spectrum of biotic and abiotic factors. A major goal of our larger project is to address concerns of the local communities regarding reef health, so we considered input from local people about the degree of fishing pressure, perceived reef degradation, and historical and current use in order to aid in site selection. Surveys were conducted during June-July of each year.

**Table 1:** List of study sites on Ulithi Atoll, Yap State, Federated States of Micronesia. Columns from left to right correspond to: the site code as it appears on Fig 2, site name, human presence (inhabited/uninhabited during the study period) as it appears on Fig 2, whether the site was located within the Ulithi lagoon or exposed directly to the ocean, the island with jurisdiction for reef and fishery management, sampling (fish transects f, benthic quadrats b), straight line distance (km) between the site and the boat launch site for the island with jurisdiction, the human population of that island, and the GPS coordinates. *UAR4 and Yea3 were ca. 15 m deep, and surveyed on SCUBA.

Jurisdiction	Pop.	Human	Exposure	Code	Site name	2012	2013	2014	Dist.	Coordinates
**Asor**	70	inhabited	oceanic	AMat	Asor Matalyoch			f,b	0.97	10° 01'25.78"N, 139°46'12.20"E
**Asor**	70	inhabited	lagoon	Asor	Asor Landing	f,b	f,b	f,b	0.00	10° 01'42.41"N, 139°45'45.67"E
**Falalop**	700	uninhabited	oceanic	Bulb	Bulbul	f	f,b	f,b	11.35	09° 55'25.72"N, 139°49'02.02"E
**Federai**	180	inhabited	lagoon	Fede	Federai Landing		f,b	f,b	0.00	09° 54'11.80"N, 139°39'19.02"E
**Federai**	180	uninhabited	lagoon	Feta	Fetabul			f,b	3.75	09° 53'02.36'N, 139° 40'49.21''E
**Falalop**	700	inhabited	oceanic	FMH	Falalop Men's House	f,b	f,b	f,b	0.29	10° 00'54.09"N, 139° 47'12.68"E
**Falalop**	700	inhabited	oceanic	FPS	For Pete's Sake			f,b	0.55	10° 01'12.03"N, 139° 47'01.67"E
**Falalop**	700	uninhabited	oceanic	GiiC	Geilob Channel	f,b			14.33	09° 56'20.81"N, 139° 54'13.97"E
**Falalop**	700	uninhabited	oceanic	Giil	Geilob Wall	f,b	f,b	f,b	15.08	09° 57'04.74"N, 139° 54'39.49"E
**Mog Mog**	120	uninhabited	lagoon	Laam	Lam		f,b		14.06	10° 02'05.15"N, 139° 35'28.94"E
**Asor**	70	uninhabited	lagoon	Lamo	Lamor		f,b		2.09	10° 02'51.00"N, 139° 45'45.00"E
**Falalop**	700	uninhabited	oceanic	Loos	Loosiyep	f,b	f,b	f,b	13.38	09° 54'49.00"N, 139° 50'25.00"E
**Falalop**	700	uninhabited	lagoon	Masi	Maas inside			f,b	7.12	09° 54'49.00"N, 139° 43'15.96"E
**Falalop**	700	uninhabited	oceanic	Maso	Maas outside			f,b	7.03	10° 00'32.39"N, 139° 43'26.83"E
**Mog Mog**	120	inhabited	lagoon	Mogc	Mog Mog Landing	f,b	f,b	f,b	0.00	10° 05'07.30"N, 139° 42'30.79"E
**Mog Mog**	120	inhabited	lagoon	Mogp	Mog Mog poison		f		1.06	10° 04'59.82"N, 139° 41'57.99"E
**Mog Mog**	120	inhabited	lagoon	Mogr	Mog Mog P. rus		f		0.00	10° 05'07.30"N, 139° 42'30.79"E
**Falalop**	700	inhabited	oceanic	OIHS	Outer Islands High School			f	0.81	10° 01'23.60"N, 139° 47'21.45"E
**Federai**	180	uninhabited	lagoon	Pig1	Piig Inside			f,b	13.88	09° 46'59.63''N, 139° 40'14.42''E
**Federai**	120	uninhabited	oceanic	Pigl	Piglelei outside		f,b		10.68	09° 59'36.00"N, 139° 37'06.00"E
**Mog Mog**	120	uninhabited	lagoon	Pota	Ptongeras		f,b		4.29	10° 04'20.00"N, 139° 40'22.00"E
**Falalop**	700	inhabited	oceanic	runS	Runway Southwest		f,b	f,b	0.25	10° 01'07.46"N, 139° 47'04.15"E
**Falalop**	700	inhabited	oceanic	runW	Runway West		f,b	f,b	0.44	10° 01'14.03"N, 139° 47'01.08"E
**Mog Mog**	120	inhabited	lagoon	Sohl	Sorghlei		f,b	f,b	3.36	10° 04'33.00"N, 139° 44'14.00"E
**Mog Mog**	120	inhabited	oceanic	Song	Sohng		f,b		13.89	10° 00'12.98"N, 139° 36'50.00"E
**Falalop**	700	inhabited	oceanic	UAR1	UAR1	f,b	f,b	f,b	0.72	10° 00'42.31"N, 139° 47'16.95"E
**Falalop**	700	inhabited	oceanic	UAR2	UAR2		f	f,b	0.57	10° 00'42.31"N, 139° 47'16.95"E
**Falalop**	700	inhabited	oceanic	UAR3	UAR3			f	0.81	10° 00'41.68"N, 139° 47'21.17"E
**Falalop**	700	inhabited*	oceanic	UAR4	UAR4			f	0.57	10° 00'42.54"N, 139° 47'18.82"E
**Federai**	180	uninhabited	lagoon	Yea2	Yealil inside			f,b	6.12	09° 51'32.83"N, 139° 37'37.61"E
**Federai**	180	uninhabited*	oceanic	Yea3	Yealil deep outside			f	6.60	09° 51'23.28"N, 139° 37'18.51"E
**Federai**	180	uninhabited	oceanic	Yeal	Yealil outside		f,b	f,b	6.60	09° 51'28.22"N, 139° 37'13.79"E
**Federai**	180	uninhabited	lagoon	YWar	Yoch Warang			f,b	10.58	09° 48'48.24''N, 139° 38'49.80''E

Sites covered a range of anthropogenic disturbances, including fishing pressure, boat use (launching, anchoring), nutrient input (human and livestock waste), and physical disturbance from World War II (dredging, physical barrier construction, use of amphibious landing craft, coral mining for construction). Site selection was based on proximity to human settlements, management (restrictions on human access to reef resources), and jurisdiction (number of human dependents in a management area). We also considered environmental factors unaffected by human influence such as exposure of reefs to deep water (e.g. oceanic or lagoonal site). Lastly, we sought broad coverage of the Atoll and nearby islands.

Due to logistical constraints and diving conditions, ‘oceanic’ sites were primarily on leeward sides of islands, but exposed to very deep water, and outside the lagoon. Because local inhabitants have preferentially occupied the more sheltered locations inside the lagoon, there were few ‘inhabited oceanic’ sites, and they were mostly limited to the Leeward side of one island (Falalop; Fig 1). This was unfortunate from an experimental design perspective, but unavoidable.

The remote location and logistical considerations necessitated the use of snorkel gear rather than scuba gear for all surveys. Over the course of the study, the same team of researchers surveyed the reef crest and the reef table in shallow sites at depths between 1.5 and 5 meters. In general, the reefs dropped off quickly below this depth, as the Atoll is surrounded by very deep water. Even the lagoonal sites dropped off rapidly to sand or deeper reefs.

### Benthic characterization

Benthic community structure was evaluated using 0.25m^2^ quadrats placed randomly on the reef crest area at each site between 1.5–5 m depth. Twenty quadrats per site each year was the modal level of effort (sample sizes for all years and all sites are provided in S1 Table). Quadrat locations were selected by using a random number generator to set the distance between quadrats and direction of swim within the reef crest corridor.

Percent cover of key organisms was determined within each quadrat (counts were used for larger mobile invertebrates and giant clams). Each quadrat was documented photographically. A total of 10 functional group categories were used to assess benthic cover: stony coral, octocorals, hydrocorals, macroalgae, algal turfs, encrusting algae, cyanobacteria, bare substrate and non-coral sessile and mobile invertebrates. Stony corals and hydrocorals were categorized into one of 12 morphological groups and identified to genus when possible (S2 Table). Instances of disease, paling, and bleaching within each quadrat were noted. Stony coral colony sizes were measured by recording maximum length, width, height, nearest live coral neighbor and coral functional group for each coral that intercepted a 50 m transect line (the same transect as used for the fish counts).

### Fish community characterization

All fish were counted along 50 m transects, in the same habitat and area (1.5–5 m depth) as the quadrats, and were identified to the species level. The transect count area was along a 50 meter long transect, and extended from the sea floor to the surface of the water column. We conducted two to three transects each year at each site. For all transects, the same diver first counted mobile fish on a 5 m wide swath, before returning along the same transect and counting cryptic benthic fishes on a 1 m wide swath. The total lengths of mobile fishes were estimated to the nearest decimeter, and nearest centimeter for cryptic benthic fishes [31]. For analysis of fish community structure, fish species were classified into one of five trophic guilds: 1. herbivores, 2. planktivores, 3. corallivores, 4. carnivores, and 5. piscivores (S3 Table lists all of the species observed on the transects and the trophic category in which they were placed). Species that have a wider trophic range (omnivores) were categorized by their main food preference according to the 5 categories mentioned above. Biomass was estimated using the published length/weight relationships most appropriate for the region [31–33]. Sharks and large rays were occasionally seen on transects, but their overall low abundance makes band transects a poor approach to estimate their actual numbers and bias their contribution to biomass [34,35]. Therefore, elasmobranchs were recorded, but not included in our biomass calculations.

### Statistical analyses

We used agglomerative hierarchical clustering (Ward’s minimum variance method; *hclust* in the R Statistical Package, [36]) based on the Bray-Curtis dissimilarity index (for benthic data) and Cao dissimilarity Index (for fish data), following published recommendations [37] to look for patterns in benthic and fish community data. We used the site groups identified in this manner as the basis for our subsequent statistical analyses. To compare biomass, abundance, and the total length of fishes between site groups,we used analysis of variance (ANOVA) to compare fish biomass, abundance and total length after checking that our data met standard assumptions of normality and homoscedasticity.

We examined the effects of anthropogenic and physical environmental factors on fish community structure using permutational multivariate analysis of variance (PERMANOVA) based on distance matrices of the fish diversity at each site. To do so, we used *adonis*, in the package *vegan* [38], which partitions distance matrices among potential sources of variation. We fit linear models to these distance matrices, and evaluated the pseudo-*F* ratios with a permutation test. The number of permutations for all of these tests was set at 999.

The model for the fish assemblages (fish ~ exposure + distance + population), was selected by comparing models with all possible combinations of the following factors and variables related to site characteristics, each stratified by year to control for potential inter-annual differences: *exposure* (lagoonal or oceanic), *distance* (distance in kilometers from the site to the village with jurisdiction), *population* (number of human inhabitants of the village with jurisdiction) and *index* (a continuous measure of the site’s orientation with respect to the prevailing northeast trade winds). Similarly, we used PERMANOVA to examine the relationship between benthic cover characteristics and the same anthropogenic and physical environmental factors used to explore fish community structure. The model selected, stratified by year to control for potential inter-annual differences, was: benthic ~ exposure + distance * population. Also using PERMANOVA, the relationship between fish community composition and benthic cover was examined with the model: fish ~ Acro_thicket + Acro_table + Mound1 + Mound2 + Branch1 + Branch1 + Encrust + Foliose + Solitary + Columnar + Montipora + Coral − Monti + Soft + Fleshy + Macroalg + Turf + CCA.

## Results

**Fig 2:**
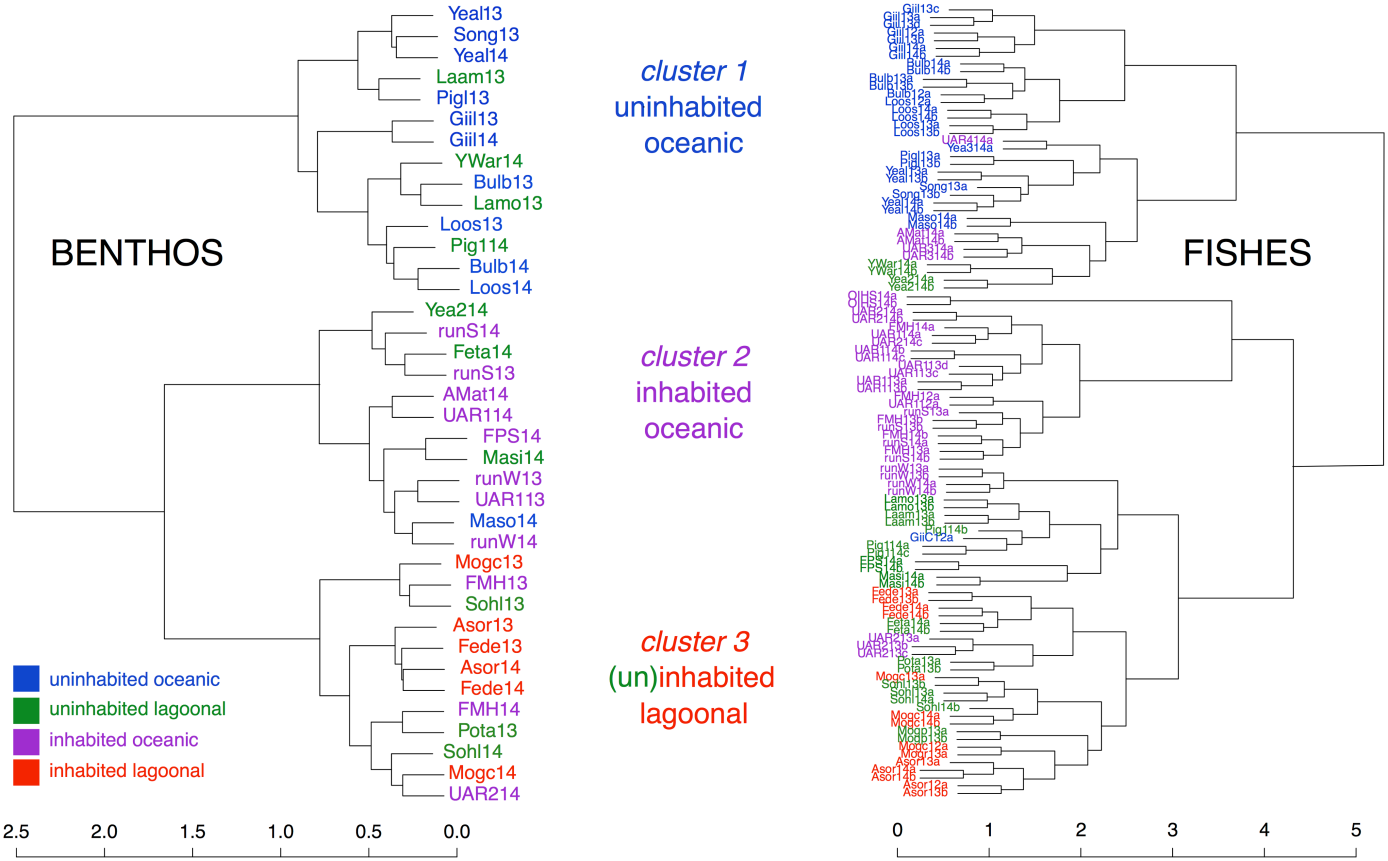
Dendrograms (Ward’s minimum variance method) of Ulithi Atoll sites based on benthic functional groups (2013 and 2014; bray-curtis dissimilarity index; left panel), and fish communities (2012, 2013 and 2014; cao dissimilarity index; right panel). The sites, ‘Mogp13a’ and ‘Mogp13b’, are, like all of the MogMog sites, ‘lagoonal’, but experience little if any fishing pressure due to the presence of a poisonous corallimorph [28].

**Fig 3:**
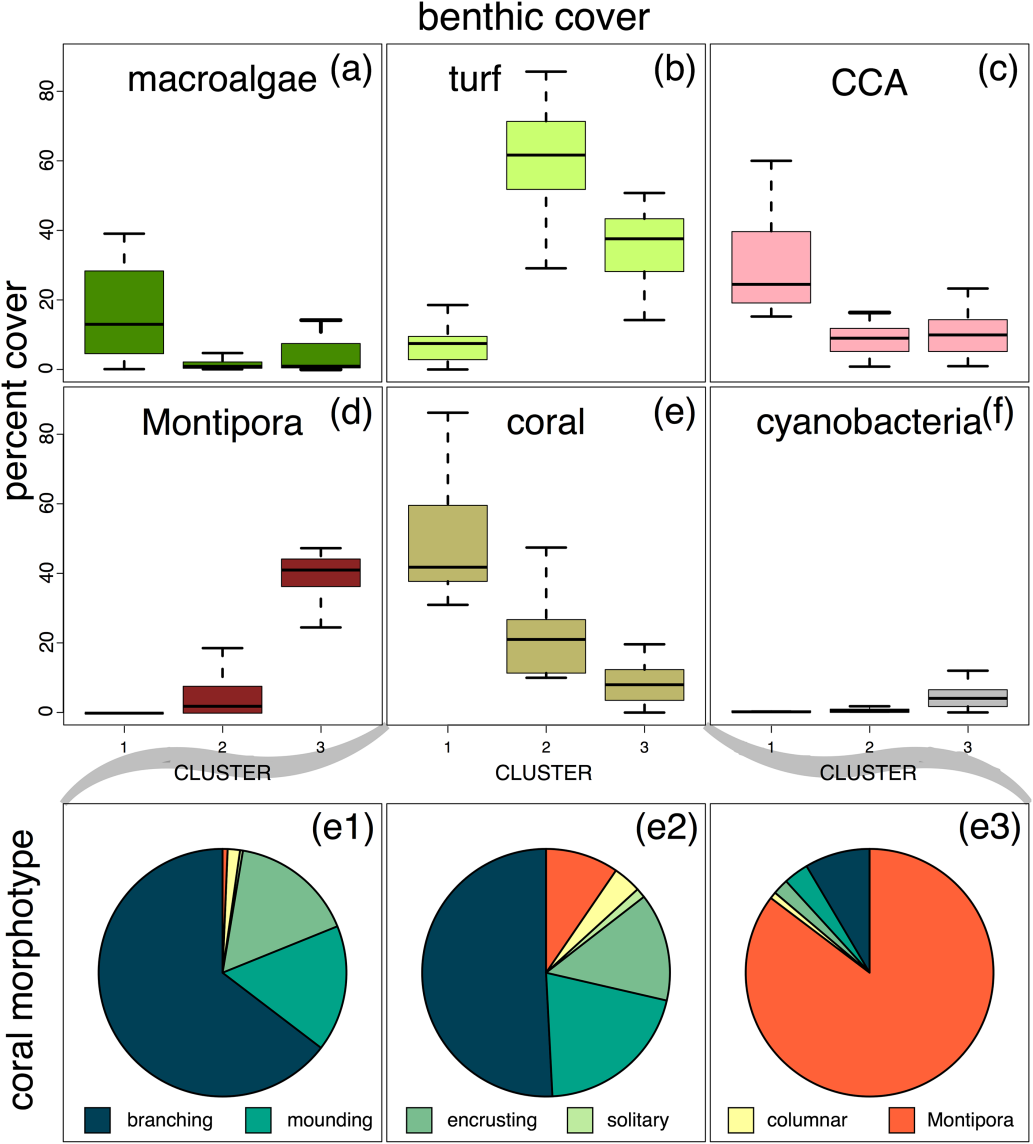
Benthic cover (% cover) compared across sites from cluster 1 (uninhabited, oceanic), cluster 2 (inhabited, oceanic), and cluster 3 (inhabited & uninhabited, lagoonal) (a-f). The relative abundance of hard coral morphotypes is shown for the 3 site clusters in e1-e3. Note that the opportunistic *Montipora* sp. is considered separately from all other stony corals for both cover and morphotype. The box-and-whisker plots show the median value (dark horizontal bar); the box length is the interquartile range, the upper whisker marks the smaller of the maximum value and quartile 3+1.5 interquartile range (IQR), and the lower whisker marks the larger of the smallest value and quartile 1–1.5 IQR. Outliers are not shown. Plots produced using the R package *graphics*, version 3.3.1.

We discovered a high prevalence of the coral identified as *Montipora* sp. [28] and reference it frequently hereafter as ‘opportunistic’ to distinguish it from species of *Montipora* that appear more slow growing (evidenced by the spatial advancement of this *Montipora* even over the course of this study). Sites with the opportunistic *Montipora* sp. were generally lagoonal sites, sites where water movement appeared low, and all sites where small boats landed frequently and/or human influence was high (near villages) (Fig 1, Table 1), identified in this paper as cluster 3 sites (Figs 2 and 3). Two other sites sampled on Falalop, farther from villages and boat landings, and exposed to deep water (RunS and FPS; Fig 1, Table 1, identified in this paper as Cluster 2 sites), had smaller amounts of this *Montipora*. Very small amounts of the same species of *Montipora* were found on the western, oceanic facing sites of Sohng and Piglelei (identified as cluster 1 sites), but none of these colonies appeared to be expanding rapidly or overgrowing neighboring coral colonies as was the case at other sites.

We conducted two separate cluster analyses: one for the benthic data, and one for the fish data. Comparing the two clusters visually (Fig 2), both dendrograms show three distinct clusters. Due to the high degree of similarity between the benthic and fish clusters, results were comparable when analyzed based on either cluster analysis. For the remainder of this paper, data are presented and discussed following the site clusters characterized by the benthic data (Fig 2 –benthos, left panel).

### Site clusters and characterization—Benthos

Most sites clustered according to their proximity to villages and high fishing activity areas, as well as relative exposure to deep water (Table 1, Fig 2); cluster 1 sites ‘uninhabited and oceanic’ were exposed to deep water, and either located outside the Atoll on neighboring islands, (e.g., Geilob Wall, ‘Giil’, Table 1), or on the Atoll (e.g. Yealil Outside, ‘Yeal’, Table 1), and were farthest from villages, with the least human impact; cluster 2 sites ‘inhabited and oceanic’ were generally near villages but were exposed to deep water (mostly the island of Falalop); and cluster 3 sites (with the exception of a few sites—see Fig 1) ‘inhabited and lagoonal’ were generally lagoonal with lower water movement, and were close to villages (e.g., Mog Mog Landing, ‘Mogc’, Table 1).

Sites in cluster 1 (uninhabited and oceanic) were arguably the ‘healthiest looking’ sites. These sites had high mean stony coral percent cover (not including the opportunistic *Montipora*) (49.3 +/- 16.6 SD), very low *Montipora* percent cover (0.35+/- 0.96 SD), high and variable macroalgal percent cover (18.3 +/- 18.6 SD), variable but low turf percent cover (8.8 +/- 8.5 SD) and high crustose coralline algae (CCA) percent cover (30.4 +/- 13.7 SD) (Fig 3). Custer 1 sites also had high coral morphological complexity (dominated by branching corals: 64.6% +/- 16.8 SD) compared with the other clusters (Fig 3).

Sites in cluster 2 (‘inhabited and oceanic’) included mainly sites on the island of Falalop, outside the Atoll lagoon (Fig 1). Most of these sites had no or low percent cover of *Montipora* sp. (4.4 +/- 5.5 SD), but one site (runway SW, Falalop) had substantial *Montipora* coverage (13.2%). Cluster 2 sites had low to moderate percent cover of other stony corals (21.6 +/- 11.1 SD) (Fig 3). Turf dominated the substrate (61.1% +/- 16.0 SD). Macroalgae (1.8% +/- 1.8 SD) and CCA cover (10.9% +/- 8.9 SD) were both low (Fig 3). Coral morphological complexity, and the prevalence of branching corals decreased as the opportunistic *Montipora* increased (Fig 3).

Sites in cluster 3 (mostly lagoonal, most of them inhabited) had low overall ‘other’ stony coral percent cover (8.0 +/- 6.2 SD), and high opportunistic *Montipora* sp. percent cover (41.6 +/- 9.2 SD). Morphological diversity was low (<15% cover of branching, mounding, columnar, and encrusting corals combined), and was dominated by the single morphotype of *Montipora* (85.3% +/- 10.1 SD). Turf algae had a relatively high percent cover (35.8 +/- 10.0 SD) whereas percent cover of macroalgae (3.6 +/- 4.2 SD) and CCA (10.6 +/- 7.1 SD) was relatively low compared to cluster 1 (Fig 3).

### Site clusters and characterization—Fishes

**Fig 4:**
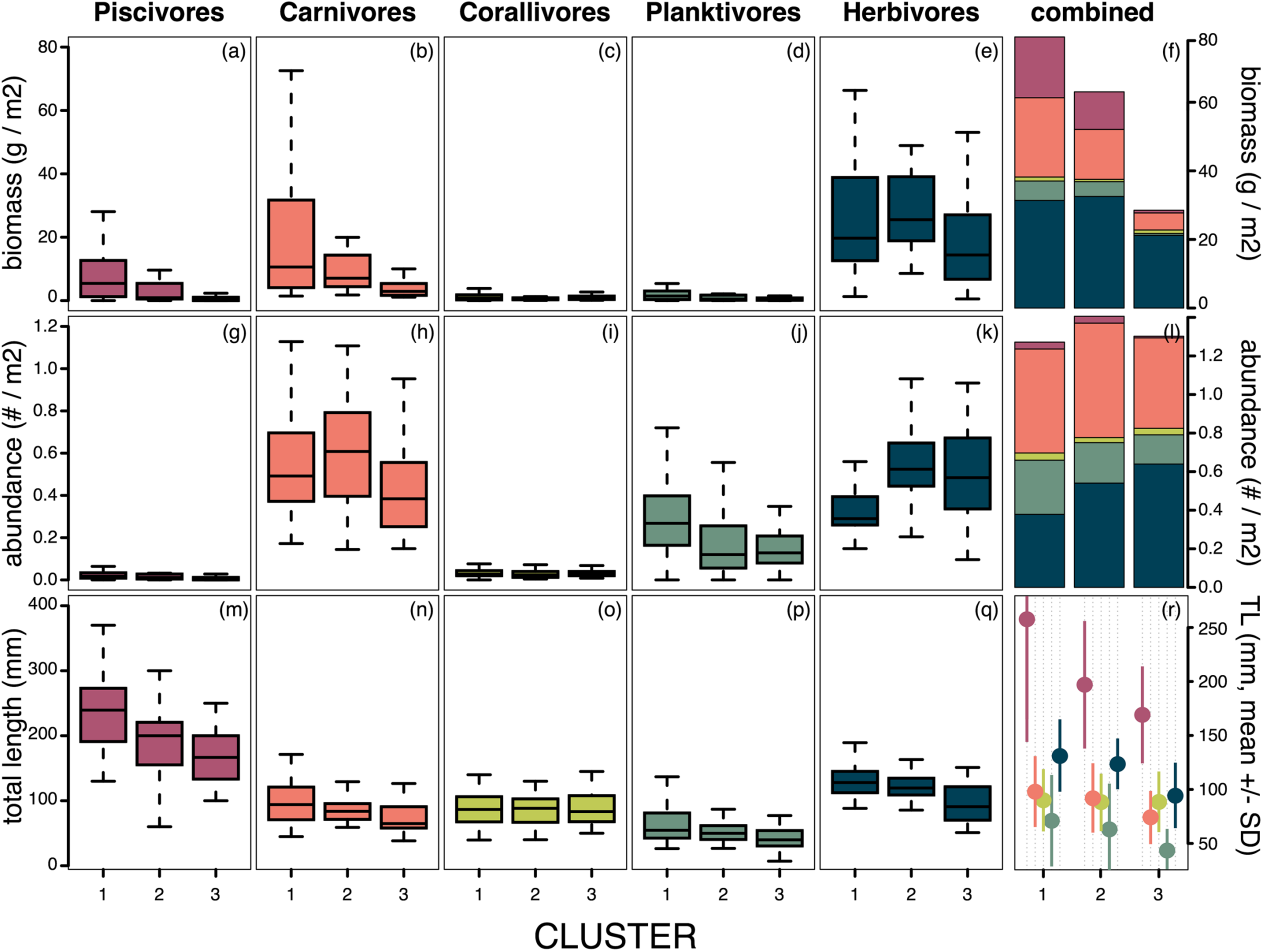
Fish biomass, abundance and size on Ulithi Atoll. Fish trophic categories (Piscivores, Carnivores, Corallivores, Planktivores, and Herbivores) are compared among site groups (cluster 1: uninhabited, oceanic; cluster 2: inhabited, oceanic; cluster 3: inhabited & uninhabited, lagoonal) for biomass (a-e), numerical abundance (g-k) and average length (TL, m-q). The stacked bar plots (f & l) show the mean values from sites within each of the 3 clusters, and the dot chart (r) compares the mean TL for fishes from trophic categories found within sites from the 3 clusters. The box-and-whisker plots show the median value (dark horizontal bar); the box length is the interquartile range, the upper whisker marks the smaller of the maximum value and quartile 3+1.5 interquartile range (IQR), and the lower whisker marks the larger of the smallest value and quartile 1–1.5 IQR. Outliers are not shown. Plots produced using the R package *graphics*, version 3.3.1.

A total of 102 (50m x 5m) fish transects were performed during our study period (2012–2014), which corresponded to a total of 22,153 counted and sized individuals, representing 276 species. Values were remarkably consistent over the years, with the mean number of fish and mean number of species per transect ranging from 208.6 to 226.9 and 34.9 to 42.6, respectively. Cluster analysis of fish community structure revealed three major groups, with the sites clustering into groups comparable to the benthic community clusters (Fig 2). The following analyses were based on the clustering of sites by benthos (Fig 2, left panel). Sites in cluster 1 (least human influence) (Fig 1, Table 1) had the highest average fish biomass at 79.0 g/m^2^ (+/- 77.1 SD, all trophic groups combined), more than double the fish biomass of cluster 3 sites (mean: 28.5 g/m^2^ +/- 24.9 SD) which were generally close to villages and within the Atoll lagoon (Fig 4) (pairwise t-test, p = 0.003 with a Bonferroni adjustment; other pairwise biomass comparisons were not significant). Cluster 2 sites were generally inhabited with greater opportunity for direct human impact, but with similar oceanic exposures as cluster 1 sites. Here, fish biomass (mean: 63.0 g/m^2^ +/- 73.2 SD) was approximately 20% lower than cluster 1 sites, but more than twice that of cluster 3 sites (Fig 4). Biomass at cluster 3 sites was lowest for all groups of fishes (28.5 g/m^2^ +/- 24.9 SD) (Fig 4).

The biomass of specific fish trophic groups varied significantly between the three clusters of sites (Fig 4, ANOVA: F_(2,99)_ = 5.805, p = 0.004). Fish biomass in cluster 1 sites was dominated by piscivores, macro-carnivores and large herbivorous species (parrotfish and surgeonfishes), while biomass in cluster 2 and cluster 3 sites was dominated by herbivorous species (Fig 4). Numerically, the clusters did not differ significantly in fish abundance (ANOVA: F_(2,99)_ = 0.5453, p = 0.581), although herbivores were more scarce in cluster 1 sites, compared to cluster 2 and cluster 3 sites (Fig 4).

Overall, the mean fish length differed significantly between the three clusters (ANOVA: F_(2,99)_ = 15.446, p<0.0001), and a pairwise t-test showed that cluster 1 (highest mean length of fishes) and cluster 2 means were significantly different from cluster 3 means (p<0.0001 and p = 0.004, respectively; cluster 1 vs. cluster 2, p = 0.194, Fig 4). With the exception of the corallivores, individual fish in the different trophic groups tended to decrease in mean length across clusters (i.e., cluster 1 vs. 2 vs. 3, Fig 4). For some groups, such as the herbivores, a greater abundance of smaller fishes appeared to maintain comparable biomass levels between cluster 1 and cluster 2 sites, and a reduction in the number of the highest trophic level piscivores was offset by an increase in the number of smaller carnivores and herbivores, especially damselfishes (Fig 4). Size structure did, however, differ among clusters, with larger piscivores and herbivores found in clusters 1 and 2, and smaller individuals in cluster 3 (Fig 4). Body size (TL, mm) differences for piscivores differed significantly among site clusters (ANOVA: F_(2,84)_ = 8.8531, p = 0.0003), also for carnivores (ANOVA: F_(2,99)_ = 5.9329, p = 0.0037), planktivores (ANOVA: F_(2,95)_ = 5.1648, p = 0.0074), and herbivores (ANOVA: F_(2,99)_ = 15.167, p<0.0001), while corallivore body size was notable for being comparable across all sites (ANOVA: F_(2,98)_ = 0.0398, p = 0.961), Fig 4).

Both human and environmental factors were correlated with differing fish community structure. PERMANOVA results support the hypothesis that fish assemblages surveyed at sites within the lagoon differed significantly from oceanic sites, and that these assemblages also differed as a function of the size of the human population with jurisdiction over that site and their distance from the inhabited island (Table 2). PERMANOVA results show that the same human and environmental factors appeared to affect benthic composition, perhaps even more directly (Table 3). The reef benthic composition differed as a function of site exposure (lagoonal vs. oceanic), distance from the nearest human community (greatest effects) and the human population size of the community with management jurisdiction. There was a significant interaction between human distance and population size (Table 3). PERMANOVA results relating aspects of the benthic cover to the composition of the associated fish communities suggest that those features that contributed positively to habitat complexity (e.g., *Acropora* sp. and the abundance of branching and mounding corals) were also related to differences in fish community structure (Table 4). The abundance of macroalgae, turf and the opportunistic species of *Montipora* were also among the benthic cover categories with a (marginally) significant effect on fish community composition (Table 4).

**Table 2:** Environmental effects and fish community structure. PERMANOVA results from the model *fish ~ exposure + distance + population*, stratified by year and terms added sequentially (first to last). Df = degree(s) of freedom, SS = Sums of Squares, MS = Mean Squares, F = F statistic obtained from the model, R^2^ = coefficient of determination, and p = probability of the obtained (model) F exceeding the predicted F statistic by chance.

	Df	SS	MS	F	R^2^	p
**Exposure**	1	9.138	9.1377	5.4253	0.09162	0.001
**Distance**	1	6.941	6.9412	4.1212	0.06960	0.001
**Population**	1	4.491	4.4910	2.6664	0.04503	0.001
**Residuals**	47	79.161	1.6843		0.79375	
**Total**	50	99.731			1.00000	

**Table 3:** Environmental effects and benthic cover composition. PERMANOVA results from the model benthic ~ exposure + distance * population, stratified by year and terms added sequentially (first to last). Df = degree(s) of freedom, SS = Sums of Squares, MS = Mean Squares, F = F statistic obtained from the model, R^2^ = coefficient of determination, and p = probability of the obtained (model) F exceeding the predicted F statistic by chance.

	Df	SS	MS	F	R^2^	p
**Exposure**	1	0.7306	0.73063	12.720	0.14100	0.001
**Distance**	1	2.0980	2.09803	36.526	0.40490	0.001
**Population**	1	0.1600	0.16005	2.786	0.03089	0.049
Distance*Population	1	0.2400	0.23997	4.178	0.04631	0.023
**Residuals**	34	1.9530	0.05744		0.37690	
**Total**	38	5.1816			1.00000	

**Table 4:** Benthic cover composition and fish community structure. PERMANOVA results from the model *fish ~ Acro_thicket + Acro_table + Mound1 + Mound2 + Branch1 + Branch2 + Encrust + Foliose + Sheet + Solitary + Columnar + Montipora + Coral-Monti + Soft + Fleshy + Macroalg + Turf + CCA*, terms added sequentially (first to last). Note that Coral-Monti refers to all stony corals not including the outbreak species of *Montipora*. Df = degree(s) of freedom, SS = Sums of Squares, MS = Mean Squares, F = F statistic obtained from the model, R^2^ = coefficient of determination, and p = probability of the obtained (model) F exceeding the predicted F statistic by chance. See S2 Table for a description of the benthic cover codes.

	Df	SS	MS	F	R^2^	p
**Acro_thicket**	1	0.26660	0.26655	1.7255	0.03268	0.026
**Acro_table**	1	0.39670	0.39673	2.5682	0.04863	0.001
**Mound1**	1	0.61540	0.61535	3.9834	0.07543	0.001
**Mound2**	1	0.38580	0.38585	2.4977	0.04730	0.004
**Branch1**	1	0.33560	0.33565	2.1728	0.04115	0.008
**Branch2**	1	0.33570	0.33572	2.1732	0.04116	0.009
**Encrust**	1	0.17310	0.17310	1.1205	0.02122	0.260
**Solitary**	1	0.36380	0.36378	2.3549	0.04458	0.004
**Columnar**	1	0.19850	0.19851	1.2850	0.02433	0.198
**Montipora**	1	0.23530	0.23533	1.5234	0.02885	0.078
**Coral-Monti**	1	0.26350	0.26345	1.7054	0.03230	0.033
**Soft**	1	0.21770	0.21767	1.4090	0.02668	0.138
**Fleshy**	1	0.18370	0.18368	1.1890	0.02252	0.240
**Macroalgae**	1	0.30610	0.30614	1.9817	0.03753	0.020
**Turf**	1	0.27890	0.27894	1.8057	0.03419	0.031
**CCA**	1	0.20250	0.20246	1.3106	0.02482	0.165
**Residuals**	22	3.39860	0.15446		0.41662	
**Total**	38	8.15750			1.00000	

## Discussion

### Linked social-ecological systems

This study has demonstrated that coral reef community structure shows distinct patterns across the scale of Ulithi Atoll, with benthic and fish communities clustering into comparable groups. The drivers of this clustering include a combination of anthropogenic factors, and reef exposure and location. Although a strong human signature may appear unusual given the low human population density, subsistence fishing, and large size of Ulithi Atoll, this study adds support to other studies demonstrating that even small populations can have substantial impacts on their ecosystems [17,39–41], resulting in a linked social-ecological system that probably goes back many centuries on Ulithi Atoll, as on other Pacific atolls [30]. An understanding of the ecological patterns and their drivers can support management planning for the communities of Ulithi and would likely be applicable to other remote, tropical islands, especially in the face of climate change and associated stressors [7,20,42].

Environmental factors that contributed to the site clustering (exposure, weather, and prevailing wind and waves) also may have contributed to historical choices for ideal village sites, linking the effects of reef location and anthropogenic impact [43–45]. Cluster 3 sites, all of which are near villages, are also located in the least exposed areas (primarily lagoonal). Inhabited lagoonal sites appear to be particularly vulnerable to reef degradation, evidenced by decreased coral cover, lower fish biomass, smaller fish sizes, and high levels of the opportunistic *Montipora* sp. (Figs 3 and 4). This could be due in part to reduced water movement and higher residence times for runoff from the villages, as well as elevated temperatures on these shallow reefs [17,46,47]. Therefore, reefs in these lagoonal habitats may exhibit reduced resiliency [7,11]. Increased fishing pressure adds to this reduced resiliency, and likely exacerbates rates of reef degradation [42,44,45]. On Ulithi Atoll, high fuel costs (keeping fishers close to shore), the loss of sailing canoes (which has resulted in greater reliance on motor boats and fishing from shore), and ‘new’ fishing methods such as night spearfishing for herbivorous fishes, have led to increased fishing pressure on reefs close to villages, likely contributing to the patterns we observed.

The opportunistic *Montipora* coral is largely associated with cluster 3 and lagoonal sites near villages and boat landings (Fig 2). While dominance by a single coral species has not been documented in other studies to the degree found here, decreased coral diversity with high coral cover has been observed in sheltered sites (notably patch reefs) on other Pacific islands [48]. Residents of Ulithi Atoll have reported decreased yield of food fish and invertebrates on *Montipora*-dominated reefs (John Rulmal Jr, personal observation). Given the importance of structural complexity for fishes and mobile invertebrates, and in particular the importance of branching corals [49], observed reductions in fishes and invertebrates at these *Montipora*-dominated sites (also reported by local fishermen) may be due to a synergistic effect of benthic structure and fishing pressure [50].

### Abiotic factors—Exposure and resiliency

While villages of the Atoll islands are positioned facing the lagoon (Asor, Mogmog, Federai), the villages of Falalop island, outside the Atoll, are an exception. Falalop is comparatively exposed and surrounded by deep water (although village sites are on the lee side of the island). Despite exposure to deep water, the sites adjacent to the villages (including Falalop Men’s House) group with cluster 3, suggesting the influence of anthropogenic factors [17]. We note however that with regard to fish community structure, Falalop Men’s House is in cluster 2, and had higher biomass than cluster 3 sites (Figs 2 and 4). This suggests an effect of abiotic factors [17], and potentially more resiliency in these sites exposed to higher water movement and deeper water [7,8,31,42].

Cluster 1 sites, westerly or southerly facing reefs and reefs outside of Ulithi Atoll (coded in blue in Fig 1), lack permanent human settlements, but are occasionally fished, especially for celebrations and community events. These sites were dominated by stony corals with high morphological complexity, extremely low or no incidence of the opportunistic *Montipora* sp., and relatively high cover of macroalgae and crustose coralline algae, consistent with data published previously [27,51]. These least impacted reefs should be carefully considered in management planning as they could serve as important recruitment pools for adjacent, more highly impacted reefs in cluster 2 and especially cluster 3 [52,53].

### Fishing pressure and site characteristics

While differences in benthic community structure were correlated with a combination of exposure to deep water and proximity to villages, fish community structure was strongly driven by distance to villages and the population size of the community with jurisdiction (Table 3). Sites near human settlements, where fishing pressure is high due to easy access, and exposure to waves and wind are reduced, clustered together based on fish diversity and biomass (lower biomass) (Fishes, cluster 3, Fig 2). These sites also had ‘lower quality’ benthic communities, perhaps exacerbated by fishing methods and depressed fish populations [4,17,40,43]. The most remote sites (Fishes, cluster 1, Fig 2, Turtle Islands, Fig 1), although included in the fishing jurisdictions of two of the four villages, had high fish biomass, lacking a strong signal of fishing pressure. In addition to lower fishing levels at these sites, fishing methods were often different, with less spearfishing and net casting (targeting herbivorous fish), and more hook and line fishing (targeting a larger diversity of fish at different trophic levels) [6,20,23,43]. Although outside the scope of this study, a historical understanding of fishing methods, landings, and traditional management is key to understanding the current patterns of coral reef community structure, and will be examined in future work [39,40,54,55].

Herbivorous fish are often the preferred catch at cluster 3 sites near villages since they can be caught from shore by spear and cast nets. Pressure on herbivorous fish was indicated by smaller sizes at these sites compared to cluster 1 sites (Fig 4). Herbivores as a functional group have been shown to have a significant role in maintaining reef habitat, as well as in facilitating reef recovery [31,56–58]. The observed reduction in herbivorous fishes is likely compromising their role in maintaining settlement and growth substrate for corals [59–61] and contributing to the decreased coral cover (not including the opportunistic *Montipora*) and morphological diversity observed at these sites. Although we found the biomass of herbivorous fish to be low at sites near villages, they were numerically abundant, with assemblages dominated by small individuals (Fig 4), consistent with trends of fishing pressure impacts on fish communities [62,63]. However, the high numbers of juveniles also indicate that these sites continue to have robust recruitment, suggesting that these sites could respond quickly to reductions in fishing pressure [64].

The correlation between benthic habitat composition and fish community structure supports previous studies suggesting that changes in one can alter the structure of the other [49,59,65–68]. Changes in community structure can be the result of destructive extraction activities, nutrient loading and physical perturbations, as well as the positive effects of habitat rehabilitation resulting from protection [62,69,70]. The opportunistic *Montipora* is a potentially important driver of community structure on these reefs into the future—possibly reducing the diversity and abundance of both corals and fishes, and affecting the productivity (from a human extraction perspective) of this system. The interplay between fish and benthic structure has important implications for management, as fishing pressure is likely to have differential impacts in these linked ecological systems.

## Conclusion

Atoll-scale patterns documented here can facilitate management planning by determining potentially resilient sites, sites under more anthropogenic pressure, and sites that are situated in locations that may predispose them to greater future degradation under multiple stressors. Finding compromised sites as well as ‘healthier’ sites can facilitate a deeper understanding of these systems and how to manage them [9]. Uninhabited, oceanic sites tended towards greater diversity and complexity of fish and benthic communities, and may be important sources of food for people while the more impacted areas are managed for recovery, so rotation strategies may be appropriate. Establishment of no-fishing zones and gear restrictions to limit pressure on specific trophic guilds, especially closer to villages, could also provide spill-over to fished areas [71,72]. Management strategies need to be carefully planned and assessed to ensure effectiveness [73,74]. Ultimately, the people of Ulithi, with enhanced knowledge about the system from studies such as this, should be supported in their management efforts within their own cultural and historical context.

The patterns in reef community structure presented here are currently being used by the people of Ulithi Atoll to develop more effective management strategies. For example, given our finding that sites that cluster as uninhabited and oceanic also have the highest densities and biomass of targeted fish, managers are utilizing those sites more during good weather and times of ample fuel in order to reduce pressure on sites near villages. Sites closer to villages–important sources of food on a regular basis–are being managed as rotating closures to enhance spillover. If communities on Ulithi and other outer islands act now by implementing traditional methods informed by scientific data, management may prove effective in a relatively short time period.

## Supporting information

S1 Table Number of benthic quadrats examined for all years and all sites. (DOCX)

S2 Table Functional group definitions for benthic community structure. (DOCX)

S3 Table Fish species observed on Ulithi Atoll fish transects 2012–2014 with trophic group designations. (DOCX)
