# Supplementary material for: Atoll-scale patterns in coral reef community structure: Human signatures on Ulithi Atoll, Micronesia
Source: PLoS One. 2017 May 10;12(5):e0177083. doi: 10.1371/journal.pone.0177083 (PMC5425048; doi:10.1371/journal.pone.0177083)
Supplement: S1 Table — (DOCX) [file pone.0177083.s001.docx]

**Supplemental Table 1.** Number of benthic quadrats examined for all years and all sites.

| **Year** | | **Site** | | **n** | | **Mode** | | |  | | |
| --- | --- | --- | --- | --- | --- | --- | --- | --- | --- | --- | --- |
| 2012 | FMH | | 36 | | 2012 | | | 36 | | |  |
| 2012 | Loos | | 48 | | 2013 | | | 18 | | |  |
| 2012 | UAR1 | | 24 | | 2014 | | | 20 | | |  |
| 2012 | UAR2 | | 48 | | all years | | | 20 | | |  |
| 2012 | Yewe | | 36 | |  | |  | | |  |  |
| 2013 | Asor | | 19 | |  | |  | | |  |  |
| 2013 | Bulb | | 19 | |  | |  | | |  |  |
| 2013 | Fede | | 19 | |  | |  | | |  |  |
| 2013 | FMH | | 20 | |  | |  | | |  |  |
| 2013 | Giil | | 18 | |  | |  | | |  |  |
| 2013 | Laam | | 19 | |  | |  | | |  |  |
| 2013 | Lamo | | 18 | |  | |  | | |  |  |
| 2013 | Loos | | 10 | |  | |  | | |  |  |
| 2013 | Mogc | | 17 | |  | |  | | |  |  |
| 2013 | Pigl | | 19 | |  | |  | | |  |  |
| 2013 | Pota | | 18 | |  | |  | | |  |  |
| 2013 | runS | | 18 | |  | |  | | |  |  |
| 2013 | runW | | 18 | |  | |  | | |  |  |
| 2013 | Sohl | | 18 | |  | |  | | |  |  |
| 2013 | Song | | 19 | |  | |  | | |  |  |
| 2013 | UAR1 | | 28 | |  | |  | | |  |  |
| 2013 | Yeal | | 18 | |  | |  | | |  |  |
| 2014 | AMet | | 20 | |  | |  | | |  |  |
| 2014 | Asor | | 20 | |  | |  | | |  |  |
| 2014 | Bulb | | 20 | |  | |  | | |  |  |
| 2014 | Fede | | 20 | |  | |  | | |  |  |
| 2014 | Feta | | 25 | |  | |  | | |  |  |
| 2014 | FMH | | 24 | |  | |  | | |  |  |
| 2014 | FPS | | 20 | |  | |  | | |  |  |
| 2014 | Giil | | 20 | |  | |  | | |  |  |
| 2014 | Loos | | 20 | |  | |  | | |  |  |
| 2014 | Masi | | 20 | |  | |  | | |  |  |
| 2014 | Maso | | 20 | |  | |  | | |  |  |
| 2014 | Mogc | | 20 | |  | |  | | |  |  |
| 2014 | Piig | | 24 | |  | |  | | |  |  |
| 2014 | runS | | 20 | |  | |  | | |  |  |
| 2014 | runW | | 20 | |  | |  | | |  |  |
| 2014 | Sohl | | 20 | |  | |  | | |  |  |
| 2014 | UAR1 | | 20 | |  | |  | | |  |  |
| 2014 | UAR2 | | 30 | |  | |  | | |  |  |
| 2014 | Yea2 | | 20 | |  | |  | | |  |  |
| 2014 | Yeal | | 20 | |  | |  | | |  |  |
| 2014 | YWar | | 25 | |  | |  | | |  |  |
