# Supplementary material for: Atoll-scale patterns in coral reef community structure: Human signatures on Ulithi Atoll, Micronesia
Source: PLoS One. 2017 May 10;12(5):e0177083. doi: 10.1371/journal.pone.0177083 (PMC5425048; doi:10.1371/journal.pone.0177083)
Supplement: S2 Table — (DOCX) [file pone.0177083.s002.docx]

**Supplemental Table 2.** Functional group definitions for benthic community structure.

| **Functional category** | **Morphological Group** | **Description** |
| --- | --- | --- |
| Stony Corals & Hydrozoans | Encrusting | Follows contour of substrate; no upright branches or unattached edges. |
|  | Sheeting/Plating | Lays against substrate. Can be layered; sheets may be thin or thick. Sheets have distinct, unattached edges. |
|  | Foliose | Layers curve upward from substrate resulting in lettuce-like appearance. |
|  | Branching 1 | Simple branching: no or few lateral arms on each projection. All branches may be same height; branches may be flattened. |
|  | Branching 2 | Complex, second-order branching: lateral arms have offshoots/branchlets. |
|  | Thicket | *Acropora* species with dense, interwoven branches; multiple individuals. |
|  | Table | *Acropora* species that sometimes have basal stem and horizontal growth with small vertical branchlets on upper surface. |
|  | Columnar | Thick columns projecting from common base with corallites. |
|  | Mounding 1 | Spherical or hemi-spherical with smooth shape; little/no vertical relief between corallites. Can be flattened (as *Symphilia*), but is thick and can produce mounds. |
|  | Mounding 2 | Two orders of complexity: overall hemispherical shape but corallites project upward >1 cm above primary mound. Provides moderate vertical relief. |
|  | Solitary | Single polyp, may be free-living |
|  | Outbreak *Montipora* sp. | Noted as own category—highly variable in growth forms, often with multiple forms simultaneously (e.g., foliose with small columns, sheeting, etc.). |
|  | *Helipora* spp. | Noted as own category with subcategory of its growth form using above forms. |
|  | *Millepora* spp. | Noted as own category with subcategory of its growth form using above forms. |
| Soft Corals | Soft coral  Leather coral | All alcyonids. |
|  | Corallimorph |  |
|  | Zooanthid |  |
| Fleshy Algae | Fleshy Brown algae |  |
|  | Fleshy Red algae |  |
|  | Filamentous Green algae |  |
|  | Fleshy Green algae |  |
| Calcareous Algae | Calcareous Green Algae |  |
|  | Calcareous Red Algae |  |
|  | Crustose Coralline Algae |  |
|  | Rhodolith |  |
| Turf | Turf | Filamentous, <2 cm length. |
| Cyanobacteria | Cyanobacteria | Noted base substrate (e.g., *Montipora*) if overgrowing coral or other living form. |
| Seagrass | Seagrass |  |
| Abiotic | Sand | > 1 cm deep |
|  | Rubble | Noted whether bare or covered with turf. |
|  | Bare calcium carbonate substrate (no corallite skeletons obvious, i.e., ‘old dead’ coral) |  |
| Non-coral Sessile invertebrates | Encrusting sponge |  |
|  | Upright sponge |  |
|  | Encrusting tunicate |  |
|  | Upright tunicate |  |
|  | Anemone |  |
| Mobile invertebrates (total # counted) | Sea cucumber |  |
|  | Sea Urchin |  |
|  | *Tridacna* |  |
|  | *Trochus* |  |
|  | Worm snail (vermetidae) |  |
