# Supplementary material for: Atoll-scale patterns in coral reef community structure: Human signatures on Ulithi Atoll, Micronesia
Source: PLoS One. 2017 May 10;12(5):e0177083. doi: 10.1371/journal.pone.0177083 (PMC5425048; doi:10.1371/journal.pone.0177083)
Supplement: S3 Table — (DOCX) [file pone.0177083.s003.docx]

**Supplementary Table 3.** Fish species observed on Ulithi Atoll fish transects 2012-2014 with trophic group designation.

| **Species** | **Family** | **Trophic group** |
| --- | --- | --- |
| *Taeniura melanospilos* | Dasyatidae | Carnivore |
| *Aetobatus narinari* | Myliobatidae | Carnivore |
| *Triaenodon obesus* | Carcharhinidae | Piscivore |
| *Nebrius ferrugineus* | Ginglymostomatidae | Carnivore |
| *Carcharhinus melanopterus* | Carcharhinidae | Piscivore |
| *Gymnothorax flavimarginatus* | Muraenidae | Piscivore |
| *Gymnothorax meleagris* | Muraenidae | Piscivore |
| *Rhinomuraena quaesita ?* | Balistidae | Carnivore |
| *Synodus binotatus* | Synodontidae | Piscivore |
| *Hemiramphus lutkei* | Hemiramphidae | Planktivore |
| *Tylosurus crocodilus* | Belonidae | Piscivore |
| *Malacanthus latovittatus* | Malacanthidae | Carnivore |
| *Gymnosarda unicolor* | Scombridae | Piscivore |
| *Carangoides orthogrammus* | Carangidae | Piscivore |
| *Caranx melampygus* | Carangidae | Piscivore |
| *Caranx sexfasciatus* | Carangidae | Piscivore |
| *Decapterus macarellus* | Carangidae | Piscivore |
| *Scomberomorus commerson* | Carangidae | Piscivore |
| *Elagatis bipinnulatus* | Carangidae | Piscivore |
| *Fistularia commersonii* | Fistulariidae | Piscivore |
| *Aulostomus chinensis* | Aulostomidae | Piscivore |
| *Pseudanthias pascalus* | Serranidae | Planktivore |
| *Anyperodon leucogrammicus* | Serranidae | Piscivore |
| *Cephalopholis argus* | Serranidae | Piscivore |
| *Cephalopholis leopardus* | Serranidae | Piscivore |
| *Cephalopholis microprion* | Serranidae | Piscivore |
| *Cephalopholis urodeta* | Serranidae | Piscivore |
| *Epinephelus fasciatus* | Serranidae | Piscivore |
| *Epinephelus hexagonatus* | Serranidae | Piscivore |
| *Epinephelus merra* | Serranidae | Piscivore |
| *Epinephelus spilotoceps* | Serranidae | Piscivore |
| *Epinephelus sp.* | Serranidae | Piscivore |
| *Epinephelus sp. 2* | Serranidae | Piscivore |
| *Gracila albomarginata* | Serranidae | Piscivore |
| *Plectropomus areolatus* | Serranidae | Piscivore |
| *Plectropomus laevis* | Serranidae | Piscivore |
| *Plectropomus leopardus* | Serranidae | Piscivore |
| *Plectropomus sp.* | Serranidae | Piscivore |
| *Variola louti* | Serranidae | Piscivore |
| *Belonoperca chabanaudi* | Grammistidae | Carnivore |
| *Aphareus furca* | Lutjanidae | Piscivore |
| *Aprion virescens* | Lutjanidae | Piscivore |
| *Lutjanus bohar* | Lutjanidae | Piscivore |
| *Lutjanus fulviflamma* | Lutjanidae | Piscivore |
| *Lutjanus fulvus* | Lutjanidae | Piscivore |
| *Lutjanus gibbus* | Lutjanidae | Piscivore |
| *Lutjanus kasmira* | Lutjanidae | Piscivore |
| *Lutjanus monostigma* | Lutjanidae | Piscivore |
| *Macolor macularis* | Lutjanidae | Piscivore |
| *Plectorhynchus orientalis* | Haemulidae | Carnivore |
| *Plectorhynchus picus* | Haemulidae | Carnivore |
| *Caesio sp.* | Caesionidae | Planktivore |
| *Caesio teres* | Caesionidae | Planktivore |
| *Pterocaesio pisang* | Caesionidae | Planktivore |
| *Pterocaesio tile* | Caesionidae | Planktivore |
| *Scolopsis lineatus* | Nemipteridae | Carnivore |
| *Gnathodentex aureolineatus* | Lethrinidae | Carnivore |
| *Monotaxis grandoculis* | Lethrinidae | Carnivore |
| *Lethrinus erythropterus* | Lethrinidae | Carnivore |
| *Lethrinus olivaceus* | Lethrinidae | Carnivore |
| *Lethrinus rubrioperculatus* | Lethrinidae | Carnivore |
| *Lethrinus harak* | Lethrinidae | Carnivore |
| *Lethrinus xanthochilus* | Lethrinidae | Carnivore |
| *Pempheris oualensis* | Pempheridae | Piscivore |
| *Kyphosus vaigiensis* | Kyphosidae | Herbivore |
| *Mulloidichthys flavolineatus* | Mullidae | Carnivore |
| *Mulloidichthys vanicolensis* | Mullidae | Carnivore |
| *Parupeneus barberinoides* | Mullidae | Carnivore |
| *Parupeneus barberinus* | Mullidae | Carnivore |
| *Parupeneus bifasciatus* | Mullidae | Carnivore |
| *Parupeneus cyclostomus* | Mullidae | Carnivore |
| *Parupeneus multifasciatus* | Mullidae | Carnivore |
| *Chaetodon auriga* | Chaetodontidae | Corallivore |
| *Chaetodon bennetti* | Chaetodontidae | Corallivore |
| *Chaetodon citrinellus* | Chaetodontidae | Corallivore |
| *Chaetodon ephippium* | Chaetodontidae | Corallivore |
| *Chaetodon kleinii* | Chaetodontidae | Corallivore |
| *Chaetodon lunula* | Chaetodontidae | Corallivore |
| *Chaetodon melannotus* | Chaetodontidae | Corallivore |
| *Chaetodon meyeri* | Chaetodontidae | Corallivore |
| *Chaetodon ornatissimus* | Chaetodontidae | Corallivore |
| *Chaetodon oxycephalus* | Chaetodontidae | Corallivore |
| *Chaetodon punctatofasciatus* | Chaetodontidae | Corallivore |
| *Chaetodon punctofasciatus* | Chaetodontidae | Corallivore |
| *Chaetodon quadrimaculatus* | Chaetodontidae | Corallivore |
| *Chaetodon reticulatus* | Chaetodontidae | Corallivore |
| *Chaetodon semeion* | Chaetodontidae | Corallivore |
| *Chaetodon trifascialis* | Chaetodontidae | Corallivore |
| *Chaetodon trifasciatus* | Chaetodontidae | Corallivore |
| *Chaetodon ulietensis* | Chaetodontidae | Corallivore |
| *Chaetodon unimaculatus* | Chaetodontidae | Corallivore |
| *Chaetodon vagabundus* | Chaetodontidae | Corallivore |
| *Forcipiger flavissimus* | Chaetodontidae | Carnivore |
| *Forcipiger longirostris* | Chaetodontidae | Carnivore |
| *Hemitaurichthys polylepis* | Chaetodontidae | Planktivore |
| *Heniochus chrysostomus* | Chaetodontidae | Carnivore |
| *Heniochus varius* | Chaetodontidae | Carnivore |
| *Centropyge bispinosus* | Pomacanthidae | Carnivore |
| *Centropyge flavissimus* | Pomacanthidae | Carnivore |
| *Centropyge loricula* | Pomacanthidae | Carnivore |
| *Centropyge vrolikii* | Pomacanthidae | Carnivore |
| *Pygoplites diacanthus* | Pomacanthidae | Carnivore |
| *Pomacanthus imperator* | Pomacanthidae | Carnivore |
| *Abudefduf vaigiensis* | Pomacentridae | Planktivore |
| *Amblyglyphidodon aureus* | Pomacentridae | Planktivore |
| *Amblyglyphidodon curacao* | Pomacentridae | Planktivore |
| *Amblyglyphidodon leucogaster* | Pomacentridae | Planktivore |
| *Amblyglyphidodon ternatensis* | Pomacentridae | Planktivore |
| *Amphiprion chrysopterus* | Pomacentridae | Planktivore |
| *Amphiprion melanopus* | Pomacentridae | Planktivore |
| *Amphiprion perideraion* | Pomacentridae | Planktivore |
| *Chromis acares* | Pomacentridae | Planktivore |
| *Chromis agilis* | Pomacentridae | Planktivore |
| *Chromis alpha* | Pomacentridae | Planktivore |
| *Chromis amboinensis* | Pomacentridae | Planktivore |
| *Chromis margaritifer* | Pomacentridae | Planktivore |
| *Chromis sp.* | Pomacentridae | Planktivore |
| *Chromis sp. Blue eye* | Pomacentridae | Planktivore |
| *Chromis ternatensis* | Pomacentridae | Planktivore |
| *Chromis viridis* | Pomacentridae | Planktivore |
| *Chromis xanthura* | Pomacentridae | Planktivore |
| *Dascyllus aruanus* | Pomacentridae | Planktivore |
| *Dascyllus reticulatus* | Pomacentridae | Planktivore |
| *Dascyllus trimaculatus* | Pomacentridae | Planktivore |
| *Dischistodus melanotus* | Pomacentridae | Carnivore |
| *Dischistodus melanotus* | Pomacentridae | Carnivore |
| *Anampses twistii* | Labridae | Carnivore |
| *Bodianus axillaris* | Labridae | Carnivore |
| *Bodianus mesothorax* | Labridae | Carnivore |
| *Cheilinus chlorourus* | Labridae | Carnivore |
| *Cheilinus digrammus* | Labridae | Carnivore |
| *Cheilinus fasciatus* | Labridae | Carnivore |
| *Cheilinus oxycephalus* | Labridae | Carnivore |
| *Cheilinus trilobatus* | Labridae | Carnivore |
| *Cheilinus undulatus* | Labridae | Carnivore |
| *Cheilio inermis* | Labridae | Carnivore |
| *Coris aygula* | Labridae | Carnivore |
| *Coris gaimard* | Labridae | Carnivore |
| *Coris variegatus* | Labridae | Carnivore |
| *Cirrhilabrus cyanopleura* | Labridae | Planktivore |
| *Epibulus insidiator* | Labridae | Carnivore |
| *Gomphosus varius* | Labridae | Carnivore |
| *Halichoeres hortulanus* | Labridae | Carnivore |
| *Halichoeres marginatus* | Labridae | Carnivore |
| *Halichoeres ornatissimus* | Labridae | Carnivore |
| *Halichoeres prosopeion* | Labridae | Carnivore |
| *Halichoeres sp.* | Labridae | Carnivore |
| *Halichoeres trimaculatus* | Labridae | Carnivore |
| *Hemigymnus fasciatus* | Labridae | Carnivore |
| *Hemigymnus melapterus* | Labridae | Carnivore |
| *Labrichthys unilineatus* | Labridae | Carnivore |
| *Labroides bicolor* | Labridae | Carnivore |
| *Labroides dimidiatus* | Labridae | Carnivore |
| *Labropsis micronesica* | Labridae | Carnivore |
| *Macropharyngodon meleagris* | Labridae | Carnivore |
| *Novaculichthys taeniourus* | Labridae | Carnivore |
| *Oxycheilinus rhodochrous* | Labridae | Carnivore |
| *Oxycheilinus unifasciatus* | Labridae | Carnivore |
| *Pseudodax moluccanus* | Labridae | Carnivore |
| *Stethojulis bandanensis* | Labridae | Carnivore |
| *Thalassoma amblycephalum* | Labridae | Carnivore |
| *Thalassoma hardwicke* | Labridae | Carnivore |
| *Thalassoma jansenii* | Labridae | Carnivore |
| *Thalassoma lunare* | Labridae | Carnivore |
| *Thalassoma lutescens* | Labridae | Carnivore |
| *Thalassoma quinquevittatum* | Labridae | Carnivore |
| *Thalassoma trilobatum* | Labridae | Carnivore |
| *Calotomus carolinus* | Scaridae | Herbivore |
| *Cetoscarus bicolor* | Scaridae | Herbivore |
| *Chlorurus frontalis* | Scaridae | Herbivore |
| *Chlorurus microrhinos* | Scaridae | Herbivore |
| *Chlorurus sordidus* | Scaridae | Herbivore |
| *Hipposcarus longiceps* | Scaridae | Herbivore |
| *Scarus altipinnis* | Scaridae | Herbivore |
| *Scarus chameleon* | Scaridae | Herbivore |
| *Scarus dimidiatus* | Scaridae | Herbivore |
| *Scarus forsteni* | Scaridae | Herbivore |
| *Scarus frenatus* | Scaridae | Herbivore |
| *Scarus globiceps* | Scaridae | Herbivore |
| *Scarus niger* | Scaridae | Herbivore |
| *Scarus oviceps* | Scaridae | Herbivore |
| *Scarus psittacus* | Scaridae | Herbivore |
| *Scarus rubroviolaceous* | Scaridae | Herbivore |
| *Scarus schlegeli* | Scaridae | Herbivore |
| *Scarus spinus* | Scaridae | Herbivore |
| *Parapercis millepunctata* | Pinguipedidae | Carnivore |
| *Zanclus cornutus* | Zanclidae | Carnivore |
| *Siganus argenteus* | Siganidae | Herbivore |
| *Siganus canaliculatus* | Siganidae | Herbivore |
| *Siganus punctatus* | Siganidae | Herbivore |
| *Siganus spinus* | Siganidae | Herbivore |
| *Acanthurus lineatus* | Acanthuridae | Herbivore |
| *Acanthurus nigricans* | Acanthuridae | Herbivore |
| *Acanthurus nigricauda* | Acanthuridae | Herbivore |
| *Acanthurus nigroris* | Acanthuridae | Herbivore |
| *Acanthurus olivaceus* | Acanthuridae | Herbivore |
| *Acanthurus pyroferus* | Acanthuridae | Herbivore |
| *Acanthurus thompsoni* | Acanthuridae | Herbivore |
| *Acanthurus triostegus* | Acanthuridae | Herbivore |
| *Acanthurus xanthopterus* | Acanthuridae | Herbivore |
| *Ctenochaetus binotatus* | Acanthuridae | Herbivore |
| *Ctenochaetus striatus* | Acanthuridae | Herbivore |
| *Ctenochaetus strigosus* | Acanthuridae | Herbivore |
| *Naso annulatus* | Acanthuridae | Herbivore |
| *Naso brachycentron* | Acanthuridae | Herbivore |
| *Naso brevirostris* | Acanthuridae | Herbivore |
| *Naso hexacanthus* | Acanthuridae | Herbivore |
| *Naso lituratus* | Acanthuridae | Herbivore |
| *Naso tonganus* | Acanthuridae | Herbivore |
| *Naso unicornis* | Acanthuridae | Herbivore |
| *Naso vlamingii* | Acanthuridae | Herbivore |
| *Zebrasoma flavescens* | Acanthuridae | Herbivore |
| *Zebrasoma scopas x flav* | Acanthuridae | Herbivore |
| *Zebrasoma scopas* | Acanthuridae | Herbivore |
| *Zebrasoma veliferum* | Acanthuridae | Herbivore |
| *Balistapus undulatus* | Balistidae | Carnivore |
| *Balistoides conspicillum* | Balistidae | Carnivore |
| *Balistoides viridescens* | Balistidae | Carnivore |
| *Melichthys niger* | Balistidae | Planktivore |
| *Melichthys vidua* | Balistidae | Planktivore |
| *Rhinecanthus rectangulus* | Balistidae | Carnivore |
| *Sufflamen bursa* | Balistidae | Carnivore |
| *Sufflamen chrysoptera* | Balistidae | Carnivore |
| *Amanses scopas* | Monacanthidae | Carnivore |
| *Ostracion meleagris* | Ostraciidae | Carnivore |
| *Aluterus scriptus* | Monacanthidae | Planktivore |
| *Cantherhines dumerilii* | Monacanthidae | Carnivore |
| *Cantherhines pardalis* | Monacanthidae | Carnivore |
| *Oxymonacanthus longirostris* | Monacanthidae | Corallivore |
| *Arothron meleagris* | Tetraodontidae | Carnivore |
| *Arothron nigropunctatus* | Tetraodontidae | Carnivore |
| *Neoniphon sammara* | Holocentridae | Carnivore |
| *Myripristis berndti* | Holocentridae | Carnivore |
| *Myripristis violacea* | Holocentridae | Carnivore |
| *Myripristis woodsi* | Holocentridae | Planktivore |
| *Sargocentron caudimaculatum* | Holocentridae | Carnivore |
| *Sargocentron diadema* | Holocentridae | Carnivore |
| *Sargocentron sp.* | Holocentridae | Carnivore |
| *Sargocentron spiniferum* | Holocentridae | Carnivore |
| *Sebastapistes cyanostigma* | Scorpaenidae | Carnivore |
| *Corythoichthys sp.* | Syngnathidae | Carnivore |
| *Caracanthus maculatus* | Caracanthidae | Carnivore |
| *Caracanthus unipinna* | Caracanthidae | Carnivore |
| *Cirrhitichthys oxycephalus* | Cirrhitidae | Carnivore |
| *Cirrhitus pinnulatus* | Cirrhitidae | Carnivore |
| *Paracirrhites arcatus* | Cirrhitidae | Carnivore |
| *Paracirrhites forsteri* | Cirrhitidae | Carnivore |
| *Cheilodipterus quinquelineata* | Apogonidae | Carnivore |
| *Chromis amboinensis* | Pomacentridae | Planktivore |
| *Chrysiptera cyanea* | Pomacentridae | Carnivore |
| *Chrysiptera leucopoma* | Pomacentridae | Carnivore |
| *Chrysiptera glauca* | Pomacentridae | Carnivore |
| *Chrysiptera sp.* | Pomacentridae | Carnivore |
| *Chrysiptera traceyi* | Pomacentridae | Carnivore |
| *Plectroglyphidodon dickii* | Pomacentridae | Carnivore |
| *Plectroglyphidodon johnstonianus* | Pomacentridae | Carnivore |
| *Plectroglyphidodon imparipennis* | Pomacentridae | Carnivore |
| *Plectroglyphidodon lacrymatus* | Pomacentridae | Carnivore |
| *Plectroglyphidodon leucozonus* | Pomacentridae | Carnivore |
| *Pomacentrus philippinus* | Pomacentridae | Planktivore |
| *Pomacentrus vaiuli* | Pomacentridae | Planktivore |
| *Stegastes fasciolatus* | Pomacentridae | Herbivore |
| *Stegastes lividus* | Pomacentridae | Herbivore |
| *Stegastes nigricans* | Pomacentridae | Herbivore |
| *Cirripectes sp.* | Blenniidae | Herbivore |
| *Ecsenius opsifrontalis* | Muraenidae | Piscivore |
| *Exallias brevis* | Blenniidae | Corallivore |
| *Gnatholepis anjerensis* | Gobiidae | Carnivore |
| *Meiacanthus atrodorsalis* | Blenniidae | Planktivore |
| *Plagiotremus tapeinosoma* | Blenniidae | Carnivore |
| *Ptereleotris evides* | Microdesmidae | Planktivore |
| *Amblygobius phalaena* | Gobiidae | Carnivore |
| *Valenciennea strigata* | Gobiidae | Carnivore |
| *Salarias sp.* | Blennidae | Herbivore |
| *Pseudochromis cyanotaenia* | Pseudochromidae | Carnivore |
| *Pseudocheilinus hexataenia* | Labridae | Carnivore |
| *Pseudocheilinus evanidus* | Labridae | Carnivore |
| *Canthigaster solandri* | Tetraodontidae | Carnivore |
| *Canthigaster valentini* | Tetraodontidae | Carnivore |
